# MR-Guided Radiotherapy for Brain and Spine Tumors

**DOI:** 10.3389/fonc.2021.626100

**Published:** 2021-03-08

**Authors:** Danilo Maziero, Michael W. Straza, John C. Ford, Joseph A. Bovi, Tejan Diwanji, Radka Stoyanova, Eric S. Paulson, Eric A. Mellon

**Affiliations:** ^1^Department of Radiation Oncology, Sylvester Comprehensive Cancer Center, Miller School of Medicine, University of Miami, Miami, FL, United States; ^2^Department of Radiation Oncology, Medical College of Wisconsin, Milwaukee, WI, United States

**Keywords:** glioblastoma, brain and spine tumors, MRI, MRgRT, radiotherapy, pseudoprogression

## Abstract

MRI is the standard modality to assess anatomy and response to treatment in brain and spine tumors given its superb anatomic soft tissue contrast (e.g., T1 and T2) and numerous additional intrinsic contrast mechanisms that can be used to investigate physiology (e.g., diffusion, perfusion, spectroscopy). As such, hybrid MRI and radiotherapy (RT) devices hold unique promise for Magnetic Resonance guided Radiation Therapy (MRgRT). In the brain, MRgRT provides daily visualizations of evolving tumors that are not seen with cone beam CT guidance and cannot be fully characterized with occasional standalone MRI scans. Significant evolving anatomic changes during radiotherapy can be observed in patients with glioblastoma during the 6-week fractionated MRIgRT course. In this review, a case of rapidly changing symptomatic tumor is demonstrated for possible therapy adaptation. For stereotactic body RT of the spine, MRgRT acquires clear isotropic images of tumor in relation to spinal cord, cerebral spinal fluid, and nearby moving organs at risk such as bowel. This visualization allows for setup reassurance and the possibility of adaptive radiotherapy based on anatomy in difficult cases. A review of the literature for MR relaxometry, diffusion, perfusion, and spectroscopy during RT is also presented. These techniques are known to correlate with physiologic changes in the tumor such as cellularity, necrosis, and metabolism, and serve as early biomarkers of chemotherapy and RT response correlating with patient survival. While physiologic tumor investigations during RT have been limited by the feasibility and cost of obtaining frequent standalone MRIs, MRIgRT systems have enabled daily and widespread physiologic measurements. We demonstrate an example case of a poorly responding tumor on the 0.35 T MRIgRT system with relaxometry and diffusion measured several times per week. Future studies must elucidate which changes in MR-based physiologic metrics and at which timepoints best predict patient outcomes. This will lead to early treatment intensification for tumors identified to have the worst physiologic responses during RT in efforts to improve glioblastoma survival.

## Introduction

Despite the potential of Magnetic Resonance image guided Radiation Therapy (MRgRT) to treat brain tumors, a recent review (1) highlighted that only one out of twenty recent studies used MRgRT to treat brain tumor patients (2). This is because MRgRT has been almost exclusively applied to treat moving tumors located in the torso, such as in the lungs (3), breast (4), pancreas (5, 6), liver (7), prostate (8) and pelvis (9). Tumor and healthy tissue in these regions can move significantly between or during treatments due to physiological motion such as respiration (3, 10, 11), digestion (12), and involuntary movements (13). Additionally, target geometry may change during treatment from tumor growth or shrinkage or patient weight loss or gain. Therefore, MRgRT has been applied to detect and compensate motion, as well as detect and compensate for daily anatomic changes with rapid radiotherapy (RT) plan updates. These implementations of MRgRT are commonly termed “adaptive radiotherapy” and are available within existing MRgRT products. Since this existing workflow adapts to anatomy, we propose that these techniques be termed “anatomic adaptive radiotherapy.”

MRI can also provide physiologic information such as tumor cellularity, vascularity, and metabolism that correlate with radiotherapy response. For example, changes in regional water mobility are detectable by diffusion weighted imaging (DWI) and are associated with increased cellularity (tumor growth) or necrosis (14, 15). Increased blood volume and flux (16) can be estimated from perfusion MRI and correlate to tumor oxygen consumption (16). Tumor extension and aggressiveness are also associated with its metabolic profile and can be estimated by magnetic resonance spectroscopy (17) (MRS). Among others, these techniques are collectively termed multiparametric MRI (mpMRI). Since changes in mpMRI during RT correlate with eventual tumor response (18–20), there is significant interest within the MRgRT community in adapting RT to mpMRI findings (21). For example, if mpMRI demonstrates that a tumor is increasingly cellular, metabolic, and angiogenic during treatment (i.e., resistant to standard therapies), should RT dose-escalation or other additional therapies be considered? When adapting RT to changes in tumor physiology, these applications can be called “physiologic adaptive radiotherapy” (PART).

Studies of physiologic changes during fractionated RT are not currently widespread because it has never been feasible before MRgRT systems to obtain mpMRI on a daily basis. It has been very difficult to obtain image data weekly due to the cost and logistics of scanning RT patients every week on diagnostic MRI scanners. Therefore, existing data of mpMRI during RT has been limited to a small number of institutions and patients and a limited number of time points (typically once or twice during a 6-week course of RT). While this data has been promising, MRgRT devices allow the possibility of obtaining mpMRI with high frequency throughout treatment to elucidate trends in tumor physiology that can be leveraged to make adaptive treatment decisions. With this in mind, this review discusses the potential use of anatomic and physiologic adaptive radiotherapy for treating brain and spine tumors with an emphasis on glioblastoma.

## Adaptive Radiotherapy for Brain Tumors

**Figure 1:**
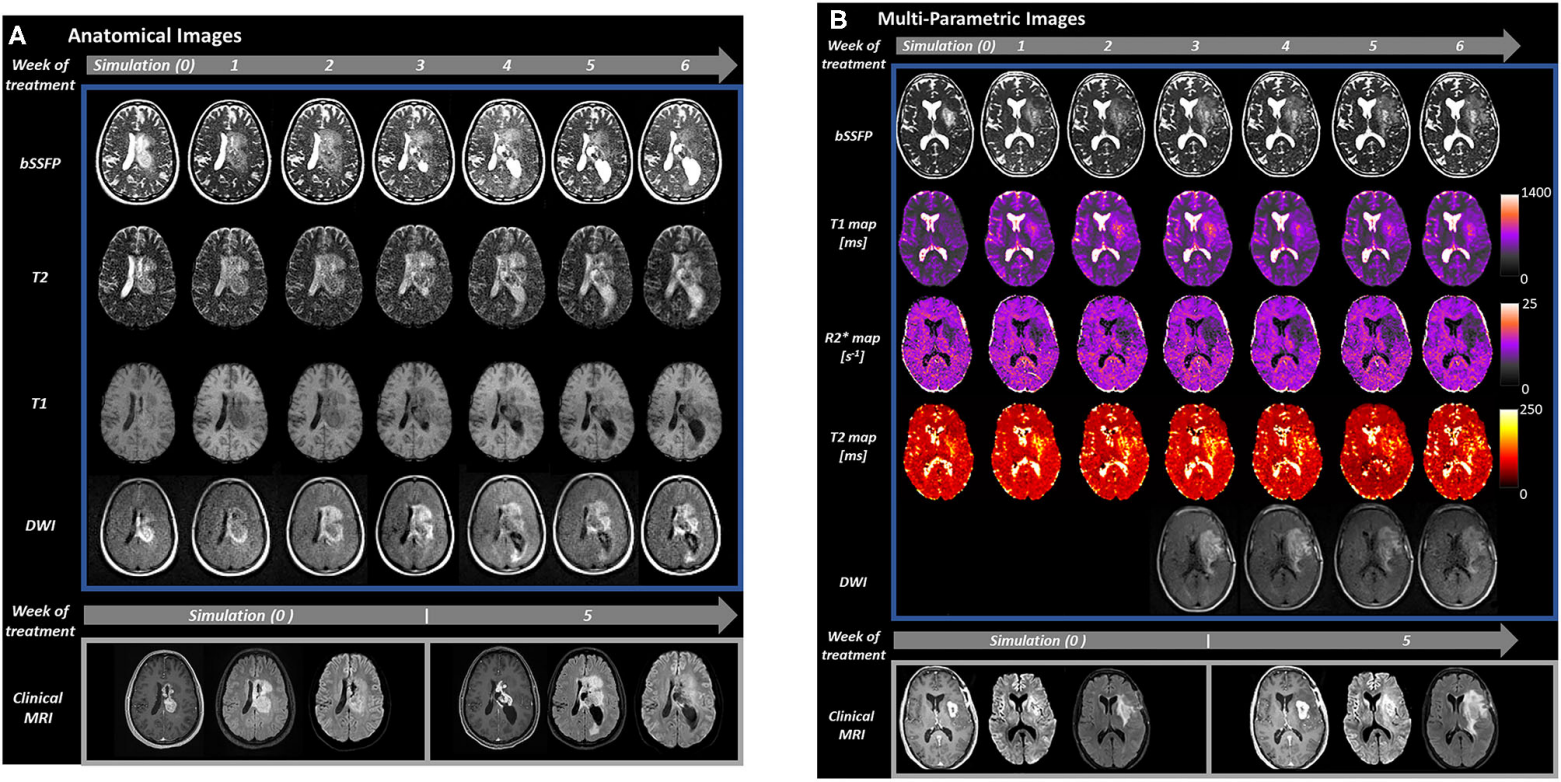
Serial MRI of two patients with glioblastoma acquired during MRgRT on the 0.35 T MRIdian (Viewray, Cleveland, OH) combination MRI and RT system at the University of Miami (top of image, blue rectangle). Imaging was obtained at simulation (week 0) and daily on MRIdian through the course of treatment, though shown weekly for simplicity (gray arrows with treatment week number). Our MRIdian workflow for glioma patients includes 20 min for daily patient setup and intensity modulated RT which includes whole brain highly T2 weighted bSSFP (1.5 × 1.5 × 1.5 mm, 128 s) for positioning, 3D couch shifts applied by the therapist analogous to non-MRI guided RT systems, and cine MRI during RT for position verification through treatment. RT is then followed by 15 min per day of additional mpMRI imaging with the patient in the same position for a total daily time of about 35 min. Comparison images are shown for each patient from a 3 T Skyra (Siemens, Erlangen, Germany) clinical scanner (bottom of image, gray rectangle) during simulation and at week 5 (RT fraction 21) of treatment. **(A)** Anatomical images (bSSFP, T2, T1, and DWI) from a 29 year old woman with a centrally located glioblastoma (IDH-1 and IDH-2 mutations negative, MGMT non-hypermethylated, H3K27M mutation negative). The patient underwent biopsy 2 weeks prior to simulation, started RT 1 week after simulation, and received 6 weeks of radiation therapy to 60 Gy in 30 fractions on the MRIdian system with concurrent temozolomide. At the bottom of the figure, the clinical scans from the left to the right-hand side are T1 post-contrast, T2 FLAIR and DWI, respectively. During week 3 of treatment, the patient's left temporal lateral ventricle became obstructed by growth of the centrally located tumor and progressive enlargement was observed. The patient became symptomatic during week 4 with headache and nausea that was controlled with dexamethasone 2 mg twice daily. After consultation with neurosurgery, the patient's radiation therapy and chemotherapy course was completed without additional intervention. The gadolinium enhancing tumor at fraction 21 had grown 7 mm outside of the gross tumor volume defined at simulation. **(B)** Multi-parametric images of a 58 year old woman with partially resected glioblastoma (IDH-1 R132H wildtype, MGMT non-hypermethylated) of the left temporal lobe with unresected portions extending into the left basal ganglia and corona radiata as shown. From top to bottom, bSSFP, T1, R2*, and T2 maps, and DWI are presented. DWI data was not available on our MRIdian system until the third week of treatment when it was added to our acquisition protocol every other day. On the bottom of the image, comparison 3 T scans at simulation and week 5 (fraction 21) from the left to the right hand side are T1 post-contrast, T2 FLAIR and DWI, respectively. This patient had progressive growth throughout treatment that was particularly prominent on fraction 21 T1 post-contrast scan (enhancing gross tumor volume margin growth of 8 mm) and R2* mapping.

Intrafraction motion is typically not a major concern for brain tumors given the use of thermoplastic masks to immobilize the patient's head and negligible physiologic motion. However, interfraction changes in tumor size can be problematic in numerous scenarios. For example, certain tumors can have rapid cyst expansion, which has been most commonly described for craniopharyngioma (22). This leads to a recommendation for weekly or bi-weekly diagnostic MRI to ensure appropriate target dose coverage and adapt RT plans offline to anatomic changes if needed. While it has not yet been reported in the literature, cysts can be monitored on an MRgRT system and RT can be adapted easily without requiring standalone diagnostic MRIs. Additionally, edema and resection cavities are visualized with default imaging on the initial version of the 0.35T MRI system (2). For example, at University of Miami during a course of conventionally fractionated RT we have used scans obtained with MRgRT to identify or rule out serious pathologies in patients with headaches during treatment, identify edema increase or decrease during RT, and reassure patients, manage steroid doses, or consult neurosurgery based on findings (e.g., Figure 1A).

### Glioblastoma

Glioblastoma is the most common cancer originating in the brain with ~12,000 new diagnoses per year in the U.S.A. and median survival about 18 months (23–25). First-line treatment for glioblastoma includes biopsy or resection followed by 6 weeks of RT with concurrent temozolomide chemotherapy and 6–12 months of continued temozolomide (26). Clinically, MRI is obtained before RT for planning and then 1 month after RT to assess early response, usually an interval of ~3 months.

### Anatomic Changes in Glioblastoma During RT

T1 post-contrast and T2-FLAIR images are typically used for determining tumor response to treatment, most commonly by applying criteria specified by Response Assessment in Neuro-Oncology (RANO) (27). Up to 49% of patients with glioblastoma demonstrate growth on T1 gadolinium-enhanced MRI acquired after the 6 weeks of standard chemoradiation treatment (28, 29). Patients with true progression of non-responding tumor continue to progress on serial MRIs and often die within 9 months (30–32). Some patients with growth on MRI after chemoradiation will stabilize or spontaneously regress without treatment modification, a condition termed pseudoprogression (30–32). This condition reflects therapy response with recruitment of blood vessels and/or necrosis and improved median survival ~38 months (28, 33). Unfortunately, no current technique reliably distinguishes true progression and pseudoprogression when these changes are present within the radiotherapy field. Therefore, RANO criteria suggest follow-up imaging over the next 3–6 months to assess whether changes spontaneously resolve without modification of therapy or continue to progress.

Consistent with these well-known changes, a recent series of 14 patients treated with MRgRT identified T2-weighted volume increases >25% in 4 patients who had been scanned daily during RT treatment delivery (34). Most growth occurred late in treatment for three of the four patients, a previously unreported finding that could hold prognostic significance. Another study observed meaningful tumor dynamic changes during chemoradiation therapy by analyzing T1 post-contrast and T2-Flair images of 62 patients with glioblastoma (35). Since the amount of gadolinium enhancement is the primary metric used currently to evaluate glioblastoma evolution, a challenge to the MRgRT community in evaluating glioblastoma changes during RT is when and how often to administer gadolinium contrast during RT; or whether to use alternative measures of tumor growth. While it is unclear whether frequent gadolinium poses risks to non-allergic subjects with normally functioning kidneys, there is significant concern about potential gadolinium deposition in the brain due to repeated administrations and unclear symptoms that may associate with gadolinium (36).

### Multiparametric MRI of Glioblastoma for Response Assessment

Existing data suggests that there is an evolution in tumor physiologic changes that occur in glioblastomas during RT. Different MRI contrasts such as T1-weighted (37), T2-weighted (38), Perfusion (39), Diffusion Weighted Imaging (DWI) (40) and proton Magnetic Resonance Spectroscopy (MRS) (41) have been investigated for early detection of glioblastoma response to treatment. Many of these techniques have been implemented or are in development on MRgRT devices, and an example is given in Figure 1B.

#### T1 and T2 and Quantitative Multi-Parametric Mapping MRI

Spin-lattice (T1) and spin-spin (T2) relaxations are mechanisms intrinsic to the tissues and measurable by MRI. The different rates of relaxation can be mapped, and quantitative measures of MRI changes can be provided. For example, quantitative multi-parametric mapping (qMPM) is a technique to obtain multiple MRI parameters in a short amount of time (42). This technique has allowed for fast and accurate mapping of different relaxation parameters such as R1 (1/T1), R2^*^ (1/T2^*^), and R2 (1/T2) and their association with glioblastoma diagnosis. A previous study showed that R1 and R2 maps identify shorter relaxation times for voxels closer than further from the tumor, which was suggested to reflect tumor invasion (43). Other studies have shown promising results for using qMPM to detect sites of future tumor progression (44) and to early detect tumor progression in patients undergoing treatment with bevacizumab (45).

There may be some benefit of these quantitative measures in assessing glioblastoma response. A recent study showed the feasibility of applying the Strategically Acquired Gradient Echo (STAGE) (46) to obtain R1, R2^*^, and Proton Density (PD) maps in patients with GBM after each fraction by using the 0.35T MRI-linac system (47). Another study used MR fingerprinting to obtain these maps using the 1.5T MRI-linac system (48). The capability of observing tumor response to treatment via its size and relaxation time variations over the course of fractionated RT is an important step toward using MRgRT to adapt glioblastoma radiation treatment.

#### Perfusion

There are two main methods for measuring perfusion with gadolinium using MRI: Dynamic Susceptibility Contrast (49) (DSC) and Dynamic Contrast Enhancement (50) (DCE). DSC is based on detecting T2^*^ signal loss due to susceptibility effects from the passage of a bolus of gadolinium contrast agent (51). This method is used for estimating hemodynamic related parameters of relative cerebral blood flow (rCBF) and relative cerebral blood volume (rCBV) (52, 53), which are reported as the most sensitive parameters for differentiating tumor progression from pseudoprogression after RT (54). Multiple post-RT studies have shown that tumor progression is associated with higher values of rCBV in comparison to pseudoprogression (19, 55, 56). Alternatively, DCE parameters are obtained by detecting signal increases from dynamic acquisition of T1-weighted images during a gadolinium bolus passage (57). The resultant signal changes are used to estimate parameters such as area under the curve (AUC) and volumetric transfer constant (K^trans^), fractional blood plasma volume (Vp) and extracellular volume (Ve) (58). The K^trans^ and AUC are the DCE-derived parameters consistently reported to be higher for recurrent gliomas when compared to radiation necrosis and pseudoprogression (59–61).

MRI perfusion derived parameters have been shown to change due to chemoradiation treatment and correlate with eventual patient outcome (62, 63). For example, CBF and K^trans^ increased 30 and 10%, respectively, when DSC and DCE data from 2 weeks after treatment completion were compared to pre-treatment data (16). Larger increases were associated with shorter patient survival when compared to patients showing smaller CBF and K^trans^ changes (16). In another study, reduction in CBV post-treatment was associated with doubling of patient survival compared with patients showing increased CBV (19). Other DCE-based parameters have also been shown to change significantly due to treatment. For example, a larger decrease on volumetric plasma volume 90th percentile histogram (VP_90%_) of DCE data acquired before and after treatment was associated with pseudoprogression when compared to true progression (−39.6 vs. −2.6%) (60). Changes in perfusion parameters have also been reported for data acquired during chemoradiation treatment. For example, patients showing tumor progression presented a significantly reduced rCBV during week three of treatment when compared to pseudoprogression patients (64). Another study acquired perfusion data weekly during chemoradiotherapy to evaluate tumor perfusion response to antiangiogenic therapy during a clinical trial (65). The MRI-linac systems can provide frequent data for evaluating perfusion parameters more frequently over the course of radiotherapy. Alternatives to gadolinium such as arterial spin labeling (ASL) (66) and intra-voxel incoherent motion (IVIM) (67) may be promising to evaluate survival of patients with gliomas (68, 69) and even daily measurements during RT to evaluate tumor response may be possible on MRI-linac systems without the added risk of exogenous contrast.

#### Diffusion

Diffusion weighted imaging (DWI) is an MRI modality capable of measuring the apparent diffusion coefficient (67) (ADC), an estimate of Brownian motion of water molecules within an imaging voxel. Water molecules in the intra-cellular environment experience a highly restricted environment, while water molecules present in the extra-cellular environment experience relatively unrestricted diffusion (70). Thus, low ADC correlates with areas of high tumor cellularity (71, 72) and aggressiveness (14, 73).

Changes in tumor ADC during post-treatment follow up images is also capable of differentiating true progression from pseudoprogression and radiation necrosis (15, 74). The rationale is that while tumor growth increases cellular density and decreases regional water mobility, a successful treatment causes the breakdown of cellular membranes of the tumor, decreases regional cellular density, and increases water mobility (18, 75). For example, Elson and colleagues reported the potential use of ADC as an early marker for responsiveness to treatment of glioblastoma. The authors analyzed ADC values from voxels within the T2/Flair volume from 52 patients and verified that elevated minimum and mean ADC values are significantly correlated to Progression Free Survival (PFS) and Overall Survival (OS) (75). Additional metrics derived from DWI such as fraction, linear, planar and spherical anisotropy have also been reported to distinguish true progression from pseudoprogression (74).

The observation of ADC over time is the base for functional Diffusion Mapping (fDM) (76), a biomarker discussed as an early detector of tumor response to treatment and survival rate (14, 77–79). For example, a previous study analyzed DWI data from 60 patients undergoing concomitant RT and temozolomide (18). The authors generated fDM maps using data acquired before, 3 and 10 weeks after the start of treatment. In their results, they showed that patients with increasing number of high ADC value voxels during treatment have a longer survival rate when compared to patients with increasing number of low ADC voxels (52.6 vs 10.9 months). The fDM technique depends on several variables related to the ADC maps generation and evaluation, such as the metric chosen and thresholding for classifying voxels showing significantly increased, decreased, or stable ADC values over time. Although previous studies showed that all of these concerns can be overcome (80), the choice and number of measurement points has still been challenging, among other reasons due to scanning time availability and patient tolerance of standalone MRIs. A practical benefit of daily MRgRT is daily mpMRI to identify the best time points for comparisons or identify trends as well as consistent scanner parameters across centers.

A longitudinal evaluation of ADC maps obtained during fractionated therapy of head and neck tumors was demonstrated by Yang et al. using the 0.35T MRI-linac system (81). The group showed that the ADC values from a ROI within responding tumor increased consistently during treatment, while the ADC values from a volume not treated (brain stem) stayed the same. We believe that further studies should be done to evaluate the feasibility of obtaining more complex DWI-based maps such as fDM and fractional anisotropy using the MRI-linac systems to show tumor early response to treatment and allow for early planning adaptation.

#### Spectroscopy

Proton magnetic resonance spectroscopy (MRS) is a non-invasive method capable of estimating the concentration of different tumor-related metabolites in the brain (82). High ratios of Choline (Cho)/N-acetyl-aspartate (NAA), Cho/normalized Creatine (nCR), Cho/normalized Choline (nCho) are known to correlate with tumor grade (83). Specifically, Cho correlates to Ki-67 index, which reflects tumor proliferation of gliomas (84, 85). A high ratio (Cho)/(NAA) has been reported as a biomarker of tumor presence and is useful for delimitating glioma extension and infiltration using MRS (17, 86, 87) and MR spectroscopic imaging (MRSI) (88, 89). Given the known correlations of MRS with tumor aggressiveness and cellularity, MRSI has been integrated into the RT planning workflow in one study to select areas for dose escalation (90). Other metrics such as the choline-to-NAA index (CNI) are also commonly investigated as potential predictors of patient outcome (41).

MRS has also been applied to detect changes of metabolites during radiotherapy treatment and to associate them with patient outcome. A previous study reported that patients showing large decreases of normalized Cho from the fourth week of treatment to 2 months post-treatment correlated with a worse median OS and PFS than patients not showing such decreases (91). Another study compared MRS data from pre-RT to data from the third week of treatment and showed that patients with stable or decreased median or mean Cho/NAA ratio showed less risk of tumor progression than patients presenting increased Cho/NAA ratios over the same period (20).

We believe that the implementation of MRS sequences is technically viable on MRI-linac devices to measure metabolism during therapy. However, to the best of our knowledge it has not been done. Such implementation would allow for a more frequent evaluation of metabolites throughout chemoradiation treatment to associate early glioblastoma response to treatment. For example, glutamate and glutamine (Glx) metabolism is altered in glioblastoma, and detection of Glx is facilitated at low field (92). Glx detected by single voxel spectroscopy at 0.5T had 2-fold increase of signal-to-noise compared to 1.5T in the brains of healthy volunteers due to collapse of the C3 and C4 Glx J-coupled resonances into a “pseudo-singlet” 2.35 ppm peak at 0.5T (93). Such implementations at 0.35 T would likely be with low resolution single voxel spectroscopy that could give additional information about pseudoprogression or true progression for PART. Conversely, on 3 T scanners, whole brain Cho/NAA ratio MRSI with 5.6 × 5.6 × 10 mm resolution acquired in 15 min has been integrated into RT planning and response tracking workflows that could be considered for adaptive RT (94, 95). MRSI could theoretically be acquired on a 1.5 T MRgRT system as well, though it is unclear whether Cho/NAA MRSI on a 1.5 T MRgRT system might have suitable resolution and spectral quality for adaptive RT.

#### Combining Different Contrasts and Modeling Radiomics

In the sections above we described results of studies associating individual MRI contrast findings to glioblastoma detection and tumor response to treatment. However, several studies showed evidence that combining different contrasts and extracting multiple parameters from MRI improves the sensitivity of predicting patient outcomes (41, 72–74). Combining radiomics metrics from multiparametric MRI to clinical variables is also an important tool for predicting tumor treatment outcome (96). This approach has also been showed to benefit from the availability of multiparametric MRI. For example, the combination of multiparametric MRI for radiomics modeling was shown to predict patient overall survival using data from before chemoradiation therapy (97). Another, study showed that combining diffusion and perfusion weighted MRI for radiomics modeling improves prediction performance when compared to a model based only on conventional MRI or clinical predictors (98). The availability of MRI data from every radiotherapy fraction allows for the inclusion of a high sampling rate temporal component into radiomics modeling.

#### Technical Challenges and Limitations

Another challenge for obtaining high quality images with MR-Linac systems is related to the relatively decreased signal to noise ratio when compared to images from higher magnetic fields (≥1.5T). Therefore, a compromise among temporal and spatial resolutions is inevitable. However, strong efforts are being applied toward developing and improving data acquisition and reconstruction strategies, such as parallel imaging and non-cartesian k-space trajectories (99). Such strategies provide for fast k-space data sampling and allow more averages of the object being imaged, resulting in higher SNR images than those obtained from standard approaches. Additionally, model-based reconstruction frameworks, such as motion-corrected and high-resolution anatomically assisted (100) and image quality transfer (101) also have been shown as alternatives for improving spatial resolution of low-resolution images.

Finally, MRgRT allows for MRI acquisition while dose is delivered, which may allow for the observation of tumor changes within a single RT fraction. For example, MRI thermometry could be used to verify tumor heating during RT with hyperthermia (102) or blood oxygen level dependent MRI could monitor the increased blood flow to tumors that occurs with carbogen inhalation (103). Such approaches may be challenging, as temporal signal variances detected during radiation delivery can be related to magnetic field drifts and susceptibility artifacts due to multi-leaf-collimator movements (104).

## Stereotactic Radiotherapy of Brain and Spine Metastases

**Figure 2:**
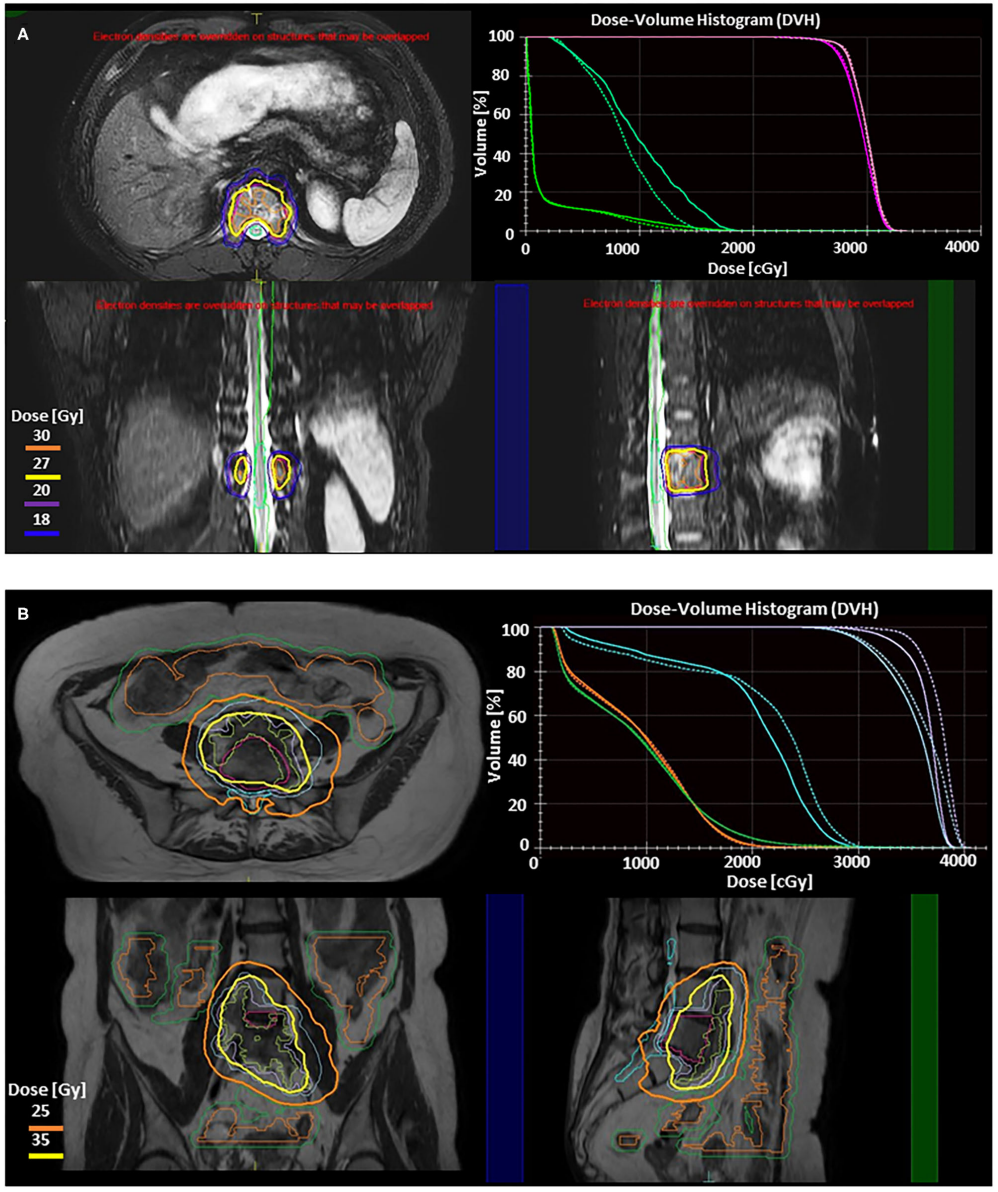
Illustration of two adaptive approaches on the 1.5 T Elekta Unity (Stockholm, Sweden) MRgRT system for stereotactic body radiotherapy (SBRT) of spine metastases. Adapt to position (ATP) is used to correct for translational shifts by adjusting beam apertures and weights without altering reference contours. Adapt to shape (ATS) accounts for all interfraction changes by re-optimizing the plan based on the MRI of the day, and requires adjustment of the target and adjacent organ at risk (OAR) contours. These treatment strategies have been described elsewhere as well as their utilization for upper abdominal SBRT (109, 110). Real-time cine MRIs acquired in perpendicular planes through the PTV center of mass are used to monitor the target during radiation delivery. **(A)** Axial, sagittal, and coronal slices from 3D T2 fat suppressed MR images from Unity showing ATP SBRT plan to T12 metastatic thyroid lesion (GTV Pink, PTV Magenta). Prescription was 27 Gy (yellow) in 3 fractions. 30 Gy (orange), 20 Gy (purple), and 18 Gy (blue) isodose lines are also shown. DVH in right upper panel compares the reference plan (solid lines) to the adaptive plan (dashed lines). This case involves a thoracic vertebrae metastasis without any extraosseous component. The target had good separation from dose limiting organs at risk without large variations in either target or OAR position or shape, making an ATP adaptive workflow optimal as recontouring is not necessary. For ATP, after the patient is positioned on the table daily MR images are obtained, fused with the reference plan, and shifts reviewed and approved by physician prior to beginning adaptation. During the adaptive process, mpMRI can be obtained simultaneously. Once a new plan is calculated, it can be reviewed by the physician, along with a verification MR and real-time cine MRI to confirm no significant intrafraction motion. The dose volume histogram (DVH) in the right upper panel demonstrates preserved target coverage with improved OAR doses for treatment. For conventionally fractionated treatments, total time on the table for patients range from 18 to 26 min, while this patient's SBRT delivery ranged from 40 to 60 min per treatment. **(B)** Axial, coronal, and sagittal slices from T2 MR images from Unity showing ATS fraction of SBRT plan to colorectal metastasis at L5 with anterior extraosseous extension. Prechemotherapy volume (blue) was prescribed 25 Gy in 5 fractions (orange) while Post-chemotherapy volume (purple) was prescribed 35 Gy in 5 fractions (yellow). DVH in right upper panel compares reference plan (solid lines) to the adaptive plan (dashed lines), demonstrating isotoxic treatment to the cauda (teal), small bowel (orange), and small bowel PRV (green) while improving coverage to both target volumes. Here the target is within close proximity to both large and small bowel. Here we use the ATS approach, with a unique parallel contouring work flow that has been described elsewhere (111). The target was rigidly fused on the daily MR, but bowel contours were different for each of five daily fractions, requiring recontouring. This allowed for maintenance of target coverage without violation of OAR constraints. ATS workflows take longer due to time required for recontouring and adapting the reference plan to not just translational shifts but new relative anatomy. For this patient the total table time ranged from 59 to 70 min.

The anatomic and physiologic adaptive radiotherapy discussed above might also be applied to short courses of radiotherapy (1–5 fraction over up to 2 weeks) commonly used in brain and spine metastases (105). In resected brain metastases, significant volume changes can happen if radiotherapy must start soon after resection (106). For example, one study showed that 9 out of 22 patients required treatment adjustments based on repeat MRI within 7 days after planning MRI and 7 out of 9 patients required adjustments in between 8 and 14 days after planning MRI (107). This suggests that anatomic adaptation might be helpful for longer fractionated courses. In the spine, bowel can migrate close to tumors within vertebral bodies, requiring anatomic adaptation to avoid mobile bowel on a daily basis (108). Examples of the anatomic adaptive workflows of MRgRT are shown in Figure 2.

While these short courses give a limited amount of time for physiologic adaptation, studies have shown that mpMRI changes correlate with response to treatment as early as 1 day and 1 week after treatment for animal models (112) and brain metastasis patients (113), respectively. Therefore, daily monitoring with MRgRT may allow for plan adaptation even in such cases. Despite the short treatment time, radiomics analysis of imaging features on the 0.35T MRgRT system were shown to correlate with outcome in pancreatic cancer (114).

## Conclusions

Novel MRgRT systems provide the first capability to perform high frequency mpMRI during conventional chemoradiotherapy of brain tumors and provide a platform for physiologic adaptive radiotherapy. The references in this manuscript suggest that combining different MRI modalities to trend tumor volume and relaxation (T1/T2/T2^*^ mapping), metabolism (MRS), hypoxia (perfusion), and cellular density (DWI) may permit a better understanding of glioblastoma response to treatment and enable dose escalated radiotherapy to portions of tumor responding inappropriately to treatment in efforts to improve patient survival. The anatomic benefits of MRI may also permit anatomic adaptation in several scenarios such as stereotactic brain and spine tumor courses.

## Author Contributions

DM, MS, and EM contributed to the elaboration of the manuscript text and figures equally. All authors equally contributed on selecting literature and topics for revision and discussion, reviewing, and organizing the manuscript.

## Conflict of Interest

The authors declare that the research was conducted in the absence of any commercial or financial relationships that could be construed as a potential conflict of interest.
